# Construction of a quality evaluation index system for nutrition nursing clinics based on the Donabedian model

**DOI:** 10.3389/fnut.2026.1867437

**Published:** 2026-09-17

**Authors:** Xing Zeng, Yu Jiang, Hui Liu, Yujia Huang, Xian Chen, Ai Li, Yajing Gu

**Affiliations:** 1 School of Nursing, Nanjing Medical University, Nanjing, China; 2 Department of Gastrointestinal and Pancreatic Surgery, The Affiliated Jiangning Hospital of Nanjing Medical University, Nanjing, China; 3 Chinese Hospital Reform and Development Institute, Nanjing University, Nanjing, China; 4 Department of Medical Oncology, The Affiliated Jiangning Hospital of Nanjing Medical University, Nanjing, China; 5 Department of Hepatobiliary and Pancreatic Surgery, Changzhou First People's Hospital, Changzhou, China; 6 Outpatient Department, The Affiliated Jiangning Hospital of Nanjing Medical University, Nanjing, China; 7 Nursing Department, The Affiliated Jiangning Hospital of Nanjing Medical University, Nanjing, China; 8 Department of Urology, The Affiliated Jiangning Hospital of Nanjing Medical University, Nanjing, China

**Keywords:** Delphi Technique, Donabedian model, indicator system, nutrition nursing clinics, quality evaluation

## Abstract

**Objective:**

To develop an evidence-based quality evaluation index system for Nutrition Nursing Clinics (NNCs) grounded in the Donabedian Structure-Process-Outcome model.

**Methods:**

A preliminary indicator system was developed through a systematic literature review, expert panel discussions, and a national survey. Utilizing the Delphi method, 17 experts participated in two rounds of consultation. Questionnaires with a 100% effective response rate in both rounds were used to collect expert opinions on the importance of indicators. Indicators were screened based on an average importance score ≥3.5 and a coefficient of variation <0.25. The Analytic Hierarchy Process (AHP) was then applied to calculate the weights for the finalized indicators.

**Results:**

The expert authority coefficients (Cr) for the two consultation rounds were 0.90 and 0.89, respectively, indicating high expert credibility. The Kendall's W coefficients for all levels of indicators were statistically significant (*P* < 0.01), demonstrating a good degree of coordination and convergence of expert opinions. Ultimately, a comprehensive quality evaluation index system for NNCs was established, comprising 3 first-level indicators, 16 second-level indicators, and 61 third-level indicators. Consistency Ratio (CR) values for all judgment matrices ranged from 0.0000 to 0.0904, confirming the logical consistency of the assigned weights.

**Conclusion:**

This study developed a preliminary, consensus-based quality evaluation index system for NNCs, representing a foundational step toward standardized evaluation in this specialty.

## 1 Introduction

With the release of the Healthy China 2030 Plan Outline (1) and the National Nutrition Plan (2017–2030) (2), clinical nutrition intervention has been elevated to a national strategic priority. The Guidelines for the Construction and Management of Clinical Nutrition Departments (2022) (3) explicitly stipulate that qualified nutrition professionals may provide standardized services, including nutrition consultation, screening and assessment, enteral nutrition preparation, medical diet formulation, and nutrition education. Nutrition Nursing Clinics (NNCs)—defined as specialized nursing services led by credentialed nutrition specialist nurses or dietitians to deliver systematic outpatient nutrition screening, assessment, diagnosis, intervention, education, and follow-up—emerged as a critical initiative for tertiary hospitals to extend chronic disease management chains and improve access to nutritional care (4). However, our recent nationwide cross-sectional survey (5) showed that among 113 sampled public medical institutions, only 37 hospitals (32.7%) had established specialized NNCs with nurse-led consultation services, of which 75.7% were initiated within the past 5 years. Concurrently, 70.3% of operational NNCs were located in Tier III Grade A hospitals, 78.4% opened only 1–3 days per week, and 75.6% served ≤ 15 patients daily. This duality of low overall penetration coupled with concentrated recent growth underscores both the rising recognition of NNCs and the urgent need to standardize service quality. Huang et al. (6) further noted that most NNCs lack clear functional positioning, leading to service overlap with clinical nutrition outpatient departments and inconsistent care quality, which hinders the standardized development of the specialty.

Existing research on nurse-led clinic quality assessment has predominantly concentrated on diabetes and wound/stoma care domains. Regarding diabetes clinics, Li et al. (7) observed a paradigm shift from conventional health education toward remote glucose management, while Heale et al. (8) employed the Donabedian framework in a multicase study across five Canadian Nurse Practitioner-led clinics, utilizing document analysis, chart audits, and interviews to systematically evaluate multimorbidity care quality and confirming that although Nurse Practitioners actively enhance care standards, workforce recruitment persists as a significant challenge; additionally, multidisciplinary diabetic foot care teams applying the Donabedian model achieved a notable 48% reduction in amputation risk (9). Within wound and stoma specialty clinics, Wang et al. (10) emphasized that quality evaluation frameworks similarly adhere to the Donabedian “structure-process-outcome (SPO)” triad, constructing nursing-sensitive quality indicators across three dimensions: nurse competency and resource allocation, standardization of assessment and intervention protocols, and clinical outcomes including healing rates and patient satisfaction, a foundation upon which Zeng et al. (11) subsequently developed preliminary multidimensional quality evaluation systems specifically targeting enteral stoma and wound care, respectively. Expanding beyond these areas, Chen et al. (12) formulated a quality evaluation index system for nurse-led Traditional Chinese Medicine outpatient clinics grounded in the Donabedian model, determining weighted indicators across all dimensions via literature synthesis, evidence grading, and a two-round Delphi expert consensus. Cumulatively, these investigations robustly affirm the broad applicability and empirical validity of the Donabedian three-dimensional quality model for performance assessment across heterogeneous nurse-led clinical settings.

Despite these advances, a dedicated, evidence-based quality evaluation system tailored to NNCs remains conspicuously absent (13). Our prior nationwide survey (5) mapped 66 quality monitoring indicators across the Donabedian framework, revealing China's first national NNC implementation profile. However, indicator adoption was highly heterogeneous: institutions prioritized structural and initial process metrics, while care continuity, interdisciplinary collaboration, and patient-reported outcomes received minimal attention. Existing nutrition-related quality assessment tools are largely tailored to inpatient settings, focusing on metrics such as enteral nutrition complication rates and duration of nutrition support, which cannot capture the unique demands of outpatient long-term follow-up and behavioral intervention in NNCs (14). Currently, NNC services in China remain in an exploratory phase, facing challenges including absent uniform service protocols, variable service content, and difficulties in objectively measuring nursing quality.

This study adopts the Donabedian SPO model as its theoretical foundation (15), given its inherent alignment with the operational logic of NNCs. The Structure dimension maps directly to foundational inputs required for NNC service delivery—including qualified nutrition specialist nurses, standardized assessment equipment, and clear institutional protocols—which are prerequisites for safe and effective care. The Process dimension corresponds to the end-to-end NNC service chain, encompassing nutritional screening, individualized intervention, health education, and longitudinal follow-up, which are critical to translating resource inputs into patient value. The Outcome dimension captures the ultimate impact of NNC services, including improvements in patients' nutritional status, quality of life, safety events, and satisfaction, aligning with the patient-centered care goals of outpatient nutrition services. The SPO framework has been widely validated in evaluating quality across diverse nurse-led outpatient settings (7–12); its systematic coverage of input, activity, and output domains avoids the one-sidedness of single-dimension evaluation approaches, making it particularly suitable for capturing the complexity of NNC services.

To address these gaps, this study explicitly targets three core research questions:

1. What constitutes a comprehensive, specialty-specific indicator pool for NNC quality evaluation? 2. How should weights be rationally assigned to indicators at all levels to reflect their relative priority? 3. Is the constructed indicator system scientifically sound and reliable for practical use? Ultimately, we aimed to develop an operational, evidence-based quality evaluation index system to support standardized NNC service delivery.

This study adopts a constructivist paradigm. Under this paradigm, the quality evaluation index system for NNCs is not conceived as a pre-existing objective standard, but rather co-constructed through the integration of literature evidence, national survey data, and the clinical experience and professional judgment of multidisciplinary experts via iterative Delphi consultations. The anonymity and iterative feedback mechanisms of the Delphi method provide an equitable platform for experts to progressively converge toward consensus, ensuring that the final system is both evidence-informed and reflective of the complexities of clinical practice.

## 2 Materials and methods

### 2.1 Establishment of the research team

The research team consisted of 10 members with clearly defined roles based on their professional expertise: the chief nurse of the department of surgery and the director of the hospital nursing department jointly oversaw the overall study design, ensured alignment with hospital policies, and provided strategic guidance throughout the research process. The head nurse of the outpatient department and the director of the clinical nutrition outpatient clinic were responsible for the on-site investigation of current NNC practices, including designing and implementing the nationwide survey on NNC status. The provincial-level clinical nutrition specialist nurse led the synthesis of policy documents and clinical guidelines and chaired the expert panel meetings to draft the preliminary indicator system. Five nurses holding a master's degree conducted the systematic literature review (searching, screening, and data extraction), managed the identification and initial contact of potential Delphi experts, assisted in organizing and implementing the two-round Delphi consultation (questionnaire distribution and collection), and performed the statistical analysis of consultation data using Microsoft Excel 2019 and Yaahp 12.6. All team members collectively participated in revising and refining the indicator system based on expert feedback across multiple rounds, and reached the final consensus through team discussions.

### 2.2 Literature search and development of the initial item pool

The initial item pool for this study was established through a rigorous, three-step process building upon our previously published national survey (5).

First, a systematic literature review was conducted. Utilizing an identical search strategy, we queried Chinese (CNKI, Wanfang, VIP) and English (Cochrane Library, PubMed, Embase, Medline, Web of Science) databases for publications spanning January 2015 to April 2024. The search employed a combination of Medical Subject Headings (MeSH) and free-text terms (e.g., “Nursing Clinics,” “Quality Indicators, Health Care,” “Nutrition Nursing”). Following screening, 19 articles were ultimately included via snowballing. These articles provided the theoretical basis for potential quality indicators.

Second, a nationwide cross-sectional survey was performed to capture current practices. Based on the insights gleaned from the aforementioned literature review, a structured questionnaire was developed and distributed nationally. This survey empirically identified 66 distinct quality monitoring indicators currently utilized across various healthcare institutions in China. These 66 indicators constituted the primary empirical dataset reflecting real-world NNC operations.

Third, the initial draft of the indicator system was synthesized. The research team integrated the scholarly evidence from the 19 included articles with the empirical findings from the 66 practice-based indicators. Guided by the Donabedian “Structure-Process-Outcome” framework, these items were deduplicated, classified, and refined through discussions by the 10-member research team to form the preliminary indicator pool for this study, which comprised 3 first-level, 13 second-level, and 85 third-level indicators.

The PRISMA flow diagram illustrating the literature screening process is reproduced in Figure 1 under the terms of the CC BY 4.0 license [Adapted from Zeng et al. (5)].

**Figure 1:**
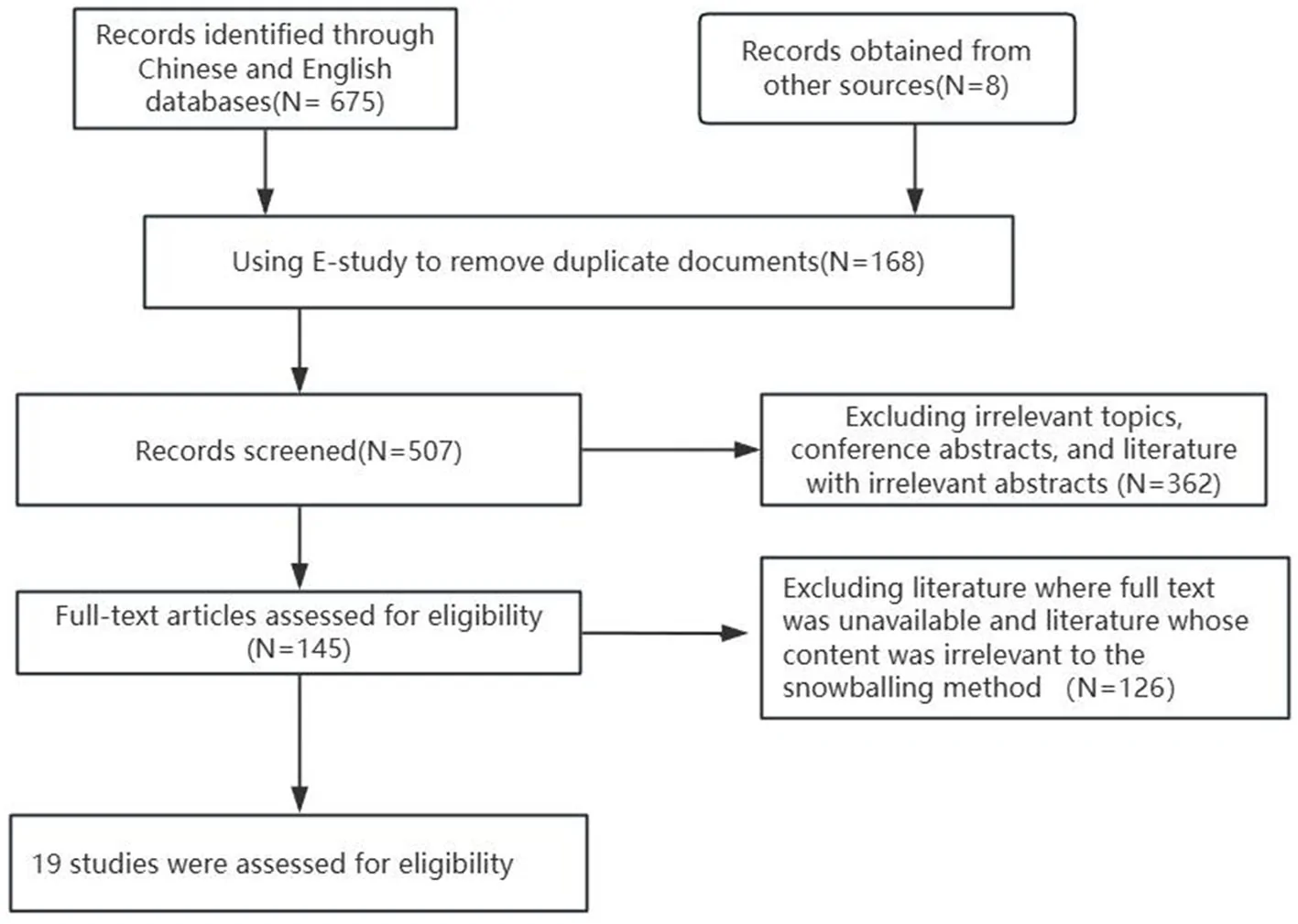
PRISMA flow diagram of literature identification, screening, and inclusion process. Reproduced from Zeng et al. (5), licensed under CC BY 4.0.

### 2.3 Delphi expert consultation

#### 2.3.1 Design of the expert consultation questionnaire

The questionnaire consisted of four parts: Consultation Instructions: Introducing the research background, objectives, significance, consultation tasks, and contact information. Expert Demographic Information Sheet: Collecting data on experts' age, education level, professional title, position, years of work experience, and main research field. NNC Quality Indicators Consultation Form: This form listed the indicators, required marking (√ or × ) to indicate whether an indicator belonged to the proposed category, a rating scale for the importance of each indicator, evaluation criteria/details, and a column for modification suggestions. The importance of each indicator was rated using a 5-point Likert scale: 5 = Very Important, 4 = Important, 3 = Moderately Important, 2 = Unimportant, 1 = Very Unimportant. Expert Authority Assessment Scale: Familiarity with the topic was self-rated on a 5-point scale from 1.0 (Very Familiar) to 0.2 (Very Unfamiliar). The basis of judgment was categorized into four types: practical experience, theoretical analysis, reference to domestic and international literature, and intuitive selection, with each assigned a quantitative value corresponding to its perceived degree of influence (Major, Moderate, Minor).

#### 2.3.2 Selection of consulting experts

(1) Number of Experts: The appropriate number of experts depends on the scale of the research project, typically ranging from 15 to 50. This study planned to select 15–20 experts via purposive sampling from tertiary Grade A general hospitals across eight regions in China.

(2) Inclusion Criteria: Employment at a tertiary Grade A hospital; Possession of a senior professional title; ≥6 years of work experience in clinical nutrition or nursing management; Bachelor's degree or higher; Interest in this research direction with a certain foundation in scientific research; Voluntary participation in this expert consultation.

(3) Exclusion Criteria: Experts who in the first round rated their familiarity as “Relatively Unfamiliar” or “Very Unfamiliar,” or whose questionnaire completion quality was deemed poor, were excluded from the second round; Experts who failed to return their opinions within the specified timeframe.

#### 2.3.3 Implementation of the expert consultation

Two rounds of Delphi expert consultation were conducted between August 2025 and January 2026. The first round was conducted from August 25 to October 31, 2025, and the second round was conducted from November 25, 2025, to January 31, 2026. A 25-day interval was deliberately scheduled between the two rounds to allow experts sufficient time for independent reflection.

*A priori* consensus criteria for item retention were set at an importance mean ≥ 3.5 and a coefficient of variation (CV) < 0.25. The Delphi process would terminate once all indicators simultaneously met these thresholds.

Questionnaires were distributed and collected via email, WeChat, and in paper form. To maintain Delphi anonymity, a quasi-anonymous approach was adopted: experts were blinded to each other's identities, and all feedback was aggregated and anonymized by the research team before redistribution.

Following the first round, the research team synthesized all expert opinions and convened a dedicated panel meeting to conduct an item-by-item deliberation. During this meeting, the necessity and rationale for modifying each indicator were thoroughly discussed, leading to a revised draft of the indicator system. When disseminating the second-round questionnaire, we explicitly informed the experts that their individual comments had been carefully reviewed and debated by the research team. To visually highlight the evolution of the indicator system and streamline the review process, all indicators that had been modified, added, or deleted based on the panel discussion were distinctly marked in red. This approach ensured that the experts could focus their re-evaluation on the substantive changes, thereby facilitating an efficient and transparent consensus-building process.

Indicator screening criteria: An indicator was retained if it simultaneously met the thresholds of an average importance score ≥ 3.5 and a coefficient of variation (CV) < 0.25. If only one criterion was met, the research team discussed the item's practicality and scientific rigor in depth before deciding on modification. Criteria for adding new items: New items were added only if suggested by experts and subsequently approved by the research team after discussion.

### 2.4 Statistical methods

#### 2.4.1 Delphi survey analysis

A database was established using Microsoft Excel 2019 for data entry and verification. Statistical analysis was performed using SPSS version 22.0. Descriptive statistics, including frequencies, means, and composition ratios, were used to characterize the data. For qualitative data, experts' written comments and suggestions from the open-ended questions were analyzed using conventional content analysis. All textual feedback was transcribed and imported into Microsoft Excel 2019, where two independent reviewers performed line-by-line coding, categorized suggestions into major themes, and identified recurring patterns. Discrepancies in coding were resolved through team discussion to reach a consensus. The synthesized qualitative findings directly informed the iterative revision of the indicator system. Expert engagement was expressed as the questionnaire response rate. Expert authority was measured by the authority coefficient (Cr), calculated as the arithmetic mean of the judgment coefficient (Ca) and the familiarity coefficient (Cs). A Cr value ≥0.7 was considered acceptable. The degree of coordination among expert opinions was assessed using Kendall's W coefficient of concordance and the coefficient of variation (CV). Kendall's W ranges from 0 to 1, typically fluctuating between 0.3 and 0.5. A high concordance coefficient, shown to be statistically significant (*P* < 0.05), indicates good coordination and high consistency among expert opinions, rendering the evaluation results credible and reliable.

#### 2.4.2 Analytic Hierarchy Process (AHP)

##### 2.4.2.1 Establishment of the Analytic Hierarchy Process (AHP) model

The AHP is a systematic methodology employed to decompose an overarching evaluation objective into hierarchical components. It typically comprises three hierarchical levels: the target layer, the criteria (or indicator) layer, and the alternatives layer. This structured approach serves as a metric for assessing the overall evaluation objective based on the alternatives layer (16). Specifically, the target layer represents the predetermined goal or research purpose of the analysis and contains a single element. The criteria layer consists of multiple sublevels encompassing the intermediate steps necessary to achieve the objective. The alternatives layer includes the various measures and decision-making options available for attaining the goal. In constructing the hierarchical model for the quality evaluation index system of NNCs, the target layer is defined as the “Quality Evaluation Index System for Clinical NNCs.” The criteria layer comprises the first- and second-level indicators of the nursing quality evaluation system, while the alternatives layer consists of the third-level indicators.

##### 2.4.2.2 Construction of the judgment matrix

A judgment matrix was employed to conduct pairwise comparisons of the relative importance of various factors at the same hierarchical level. The mean difference method of importance scores was utilized to determine the proportional scale for pairwise comparison, particularly suitable for models with multiple levels and a large number of constituent elements (16). Based on the experts' importance ratings obtained from the final round of the Delphi questionnaire survey, the differences between the mean importance scores of each pair of indicators were calculated to construct the judgment matrix. In the judgment matrix A, each element Aij represents the comparative value of the importance of the i-th indicator relative to the j-th indicator. The matrix follows the rules that Aij > 0, the diagonal elements are unity (Aii = 1), and the off-diagonal elements are reciprocals of each other (Aji = 1/Aij) (17) (Table 1).

**Table 1:** Proportional scale.

Evaluation value of indicator i relative to j	Degree of importance of indicator i relative to j	Mean difference of importance scores (Ai–Aj)
1	Equally important	0
2		0 < Ai**–**Aj ≤ 0.25
3	Moderately more important	0.25 < Ai–Aj ≤ 0.5
4		0.5 < Ai–Aj ≤ 0.75
5	Strongly more important	0.75 < Ai–Aj ≤ 1.0
6		1.0 < Ai–Aj ≤ 1.25
7	Very strongly important	1.25 < Ai–Aj ≤ 1.5
8		1.5 < Ai–Aj ≤ 1.75
9	Extremely important	Ai–Aj > 1.75

##### 2.4.2.3 Calculation of indicator weights and consistency test

###### 2.4.2.3.1 Priority vector and weight calculation.

The priority vector (i.e., local weight vector) for each judgment matrix was derived using the geometric mean method (root-squared method) (17). Briefly, for a judgment matrix of order n, the computation involved three steps: 1. calculating the product of all elements in each row; 2. computing the n-th root of each product to obtain an unnormalized priority vector; and 3. normalizing the vector such that the sum of all elements equals 1.0, thereby yielding the local weight of each indicator within its hierarchical cluster. Detailed computational formulas for this process are provided in the cited methodological literature (17).

###### 2.4.2.3.2 Composite weight calculation.

Given the hierarchical structure of the NNC quality evaluation index system, the composite weight of a lower-level indicator accounts for both its own local weight and the weights of its higher-level parent indicators. Specifically, the composite weight of a third-level indicator was calculated by multiplying its own local weight by the local weights of its corresponding second-level and first-level parent indicators. This hierarchical multiplication ensured that the sum of all composite weights equaled 1.0, reflecting each indicator's relative contribution to the overarching evaluation objective.

###### 2.4.2.3.3 Consistency test.

To assess the logical consistency of the pairwise comparisons, the maximum eigenvalue (λ_max_) was first calculated for each judgment matrix, from which the consistency index (CI) was derived. The CI value was then referenced against the random consistency index (RI) for the corresponding matrix order, with standard RI values adopted from the literature (18) and also presented in Table 2 of this study. The consistency ratio (CR) was computed as CR = CI / RI. A CR value of less than 0.10 was considered indicative of satisfactory consistency (16). All calculations were performed using Microsoft Excel 2019 and Yaahp 12.6 software.

**Table 2:** Random consistency index (RI) values.

Order (m)	1	2	3	4	5	6	7	8	9	10
RI	0	0	0.52	0.89	1.12	1.26	1.36	1.41	1.46	1.49
Order (m)	11	12	13	14	15	16	17	18	19	20
RI	1.52	1.54	1.56	1.58	1.59	1.5943	1.6064	1.6133	1.6207	1.6292
Order (m)	21	22	23	24	25	26	27	28	29	30
RI	1.6358	1.6403	1.6462	1.6497	1.6556	1.6587	1.6631	1.6670	1.6693	1.6724

## 3 Results

### 3.1 Demographic characteristics of the expert panel

A total of 17 senior experts were purposively selected from tertiary Grade A general hospitals across eight geographically diverse regions of China, including Nanjing, Hangzhou, Zigong, Kunming, Changzhou, Zhangjiagang, Suzhou, and Ningxia. This nationwide distribution ensured a comprehensive representation of clinical practices and administrative perspectives pertinent to NNCs. Detailed demographic profiles are systematically summarized in Table 3.

**Table 3:** Demographic characteristics of the Delphi expert panel.

Characteristic	Category	*n* (%)
Gender	Female	17 (100.0)
Age (mean ± SD)	42.5 ± 6.7 years (range: 35–60)	—
Age group	30–39 years	6 (35.3)
	40–49 years	9 (52.9)
	50–59 years	1 (5.9)
	≥60 years	1 (5.9)
Professional title	Full senior	3 (17.6)
	Associate senior	14 (82.4)
Professional position	Department director	1 (5.9)
	Director of nursing dept	2 (11.8)
	Division head nurse	2 (11.8)
	Ward head nurse	7 (41.2)
	Other clinical frontline experts	5 (29.4)
Educational background	Bachelor's degree	11 (64.7)
	Master's degree	5 (29.4)
	Doctoral degree	1 (5.9)
Years of experience (mean ± SD)	14.2 ± 9.3 years (range: 6–41)	—
Years of experience group	6–10 years	9 (52.9)
	11–20 years	3 (17.7)
	>20 years	5 (29.4)
Primary work area	Clinical nutrition	7 (41.2)
	Nursing management	5 (29.4)
	Clinical nutrition + nursing management	5(29.4)
Expert category	Nurse	16 (94.1)
	Physician	1 (5.9)
Master's supervisor	Yes	3 (17.6)
	No	14 (82.4)

### 3.2 Level of engagement and authority of the experts

A total of 17 eligible experts were invited, and all 17 agreed to participate, yielding a response rate of 100%. In the first round, 11 experts provided 37 distinct comments (after merging identical opinions from the original questionnaire records); in the second round, 10 experts provided 89 distinct comments. The substantially higher number of comments in the second round reflects experts' deepened engagement in refining evaluation details (e.g., calculation formulas, data sources, and performance thresholds). These were synthesized into 7 (Round 1) and 5 (Round 2), major revision categories, with details provided in Supplementary Tables S1, S2. This high volume of constructive feedback indicates a strong level of engagement and interest in the study. The authority coefficients (Cr) for the experts were 0.90 in the first round and 0.89 in the second round, demonstrating a high degree of expert authority and lending credibility to the results.

### 3.3 Degree of coordination of expert opinions

The Kendall's W coefficients of concordance for both rounds of consultation were statistically significant (*P* < 0.01), indicating a good level of agreement and consistency among the expert panel, and thus strong reliability of the results. The specific values are presented in Table 4.

**Table 4:** Expert opinion coordination degree and significance test.

Index	Round 1				Round 2			
	Coordination coefficient (W)	χ^2^	Degree of freedom (df)	*P*	Coordination coefficient (W)	χ^2^	Degree of freedom (df)	*P*
Primary index	0.327	11.128	2	0.0038	0.505	17.176	2	0.0002
Secondary index	0.335	68.41	12	0	0.441	120.066	16	< 0.0001
Three-level index	0.392	560.062	84	0	0.405	730.167	106	< 0.0001

### 3.4 Results of the expert consultation

The preliminary indicator pool was established through a rigorous, multi-source approach grounded in the Donabedian SPO framework. Integrating evidence from a systematic literature review, a nationwide questionnaire survey of 37 tertiary hospitals with operational NNCs across China, and consensus discussions among a 10-member multidisciplinary research team (including nursing managers, clinical nutrition specialists, and methodology experts), we initially developed a framework comprising 3 first-level indicators, 13 second-level indicators, and 85 third-level indicators.

First-round changes: Following the first round of Delphi consultation, revisions were made based on expert feedback and research team deliberation. At the secondary level, the number increased from 13 to 17 through splitting broad system-related items into discrete sub-indicators to improve granularity. At the tertiary level, the original 85 indicators were first evaluated against the pre-specified retention criteria (mean importance score ≥3.5 and coefficient of variation < 0.25). All 85 indicators scored 4.18–5.00; however, two were removed: 1.4.7 (CV = 0.284, exceeding the < 0.25 threshold) and 3.1.2 (Expert 11 suggested replacing complication incidence with improvement rate; team confirmed misplacement in “Patient Outcomes”). After these removals, 83 indicators remained. Through splitting broad indicators and adding new ones per expert suggestions, the tertiary pool expanded from 83 to 107 (net +24). Major modifications for added, deleted, and revised indicators in this round are provided in Supplementary Table S1.

The second round yielded an even stronger consensus: all indicator importance scores ranged from 4.00 to 5.00, with CVs between 0.00 and 0.243, confirming high consistency in expert opinions. Following this round, iterative optimization was implemented based on expert feedback, with core revisions as follows: Structural Optimization: The number of secondary indicators was streamlined from 17 to 16 by merging overlapping items. Crucially, NNC Quality Improvement and Collaboration were reclassified from outcome measures to the Process dimension to strictly align with the SPO framework logic. Content Streamlining: Following this round, 46 tertiary indicators were consolidated or removed based on expert feedback and team deliberation (primarily through merging redundant items, i.e., integrating multiple discrete indicators into core composite indicators), ultimately yielding 61 tertiary indicators. Indicator Refinement: The patient and family compliance assessment (2.1.4) was refined by replacing the original formula with flexible, clinically relevant methods (e.g., patient diaries, follow-up statements, dispensing records, or the Morisky scale), addressing expert concerns about the impracticality of the initial approach. Key revision logs are provided in Supplementary Table S2.

Through this rigorous refinement process, the final NNC quality evaluation system was constructed, comprising 3 first-level indicators, 16 second-level indicators, and 61 third-level indicators. The weights for primary and secondary indicators are summarized in Table 5. The complete list of all 61 third-level indicators, including their evaluation criteria, importance scores, and specific weights, is provided in Supplementary Table S3.

**Table 5:** Weights and psychometric characteristics of indicators in the final quality evaluation system.

Indicator	Importance score (Mean ±SD)	Coefficient of variation	Local weight	Composite weight
1 Structure	4.765 ± 0.437	0.0918	0.637	—
1.1 Human resources	4.882 ± 0.332	0.068	0.327	0.2083
1.2 Personnel assessment and training	3.765 ± 0.752	0.1999	0.069	0.0439
1.3 Environment and facilities	4.941 ± 0.243	0.0491	0.4119	0.2624
1.4 System	3.588 ± 0.795	0.2216	0.0386	0.0246
1.5 Work standards	3.941 ± 0.899	0.2282	0.0988	0.0629
1.6 Standardized processes	3.706 ± 0.849	0.2291	0.0548	0.0349
2 Process indicators	4.000 ± 0.866	0.2165	0.1047	—
2.1 Assessment	4.941 ± 0.243	0.0491	0.2532	0.0265
2.2 Nutrition nursing intervention	4.765 ± 0.437	0.0918	0.1537	0.0161
2.3 Health education	4.059 ± 0.899	0.2216	0.0545	0.0057
2.4 Patient records and follow-up management	4.941 ± 0.243	0.0491	0.2532	0.0265
2.5 Nursing clinic quality improvement	4.941 ± 0.243	0.0491	0.2532	0.0265
2.6 Collaboration	3.706 ± 0.849	0.2291	0.0323	0.0034
3 Outcome indicators	4.294 ± 0.920	0.2141	0.2583	—
3.1 Patient outcomes	4.118 ± 0.857	0.2083	0.1143	0.0295
3.2 Occurrence of safety (Adverse) events	4.824 ± 0.393	0.0815	0.4067	0.1051
3.3 Satisfaction	3.882 ± 0.857	0.2209	0.0723	0.0187
3.4 Health efficiency and benefit indicators	4.824 ± 0.393	0.0815	0.4067	0.1051

The Analytic Hierarchy Process (AHP) was employed to calculate indicator weights based on expert importance ratings. The CR values for all judgment matrices ranged from 0.0000 to 0.0904, with the highest value observed in the tertiary matrix under indicator 3.2 (Occurrence of Safety/Adverse Events). All CR values were below the 0.100 threshold, confirming the logical consistency of the weight distribution. Detailed AHP calculation matrices and consistency test results are provided in Supplementary Tables 1 and 2.

## 4 Discussion

### 4.1 Methodological rigor of the indicator system

This study adhered to internationally recognized Delphi and AHP protocols to ensure the rigor of the indicator development process, while explicitly delineating the scope of these methodological checks to address potential overstatements. Grounded in the Donabedian SPO framework, the system provides a logically closed structure encompassing resource inputs, service processes, and patient outcomes. The 17 participating senior experts, with a mean of 14.2 years of relevant experience, yielded authority coefficients (Cr = 0.89–0.90) well above the acceptable threshold of 0.7, confirming the credibility of the opinion sources. The statistically significant Kendall's W coefficients (all *P* < 0.01) indicated strong convergence of expert opinions after two rounds of consultation. For weight calculation, the overall consistency ratio (CR = 0.050) of the AHP judgment matrices was well below the 0.10 threshold, verifying the logical coherence of pairwise comparisons among experts. Notably, while this CR value confirms the internal consistency of the AHP model, the derived weights reflect expert consensus on theoretical priorities rather than empirically validated resource allocation efficiencies. Their alignment with real-world clinical outcomes and operational needs warrants validation through prospective pilot testing and multi-center trials.

### 4.2 Content analysis: comprehensiveness, specialty specificity, and critical comparison

The constructed indicator system demonstrates three interrelated strengths: comprehensive dimensional coverage, logically coherent structuring, and deep alignment with the distinctive scope of nutrition nursing practice. Rather than merely replicating generic outpatient nursing quality frameworks, our system responds to specific gaps identified in the nutrition care quality indicator literature.

Comprehensiveness within the SPO framework. Grounded in Donabedian's SPO model (19), the system achieves closed-loop coverage across resource inputs, service activities, and patient outcomes, comprising 3 primary, 16 secondary, and 61 tertiary indicators. A seminal systematic review by Moick et al. (20) 822 nutrition care quality indicators (QIs) from 46 included studies, mapping them into 19 themes. That review revealed three major weaknesses: (1) unclear development processes, (2) missing linkages between structure/process indicators and outcomes, and (3) inadequate stakeholder involvement. The review further reported that among 822 QIs, process indicators dominated (49%), while structural indicators were under-represented (23%) (20). Our two-round Delphi process with 17 senior experts directly mitigates these limitations, providing a transparent development trajectory. Notably, the Structure dimension dominated the composite weight distribution (0.637), underscoring expert consensus that foundational inputs are the prerequisite for quality care—a prioritization often neglected in prior frameworks (19, 20). This weighting aligns with Donabedian's proposition that structure is the prerequisite for quality processes (21), while acknowledging that the structure-outcome relationship is probabilistic. The Outcome dimension (composite weight 0.2583) balances patient outcomes, safety, satisfaction, and efficiency/benefit, providing a comprehensive measure of the ultimate service value (19).

Specialty specificity through concrete nutrition-sensitive indicators. The system's distinctiveness lies in the granular, nutrition-specific indicators embedded within each level, addressing the paucity of nutrition-sensitive measures in prior literature:

**Structure level:** We incorporated “*qualification requirements for NNC staff”* (1.1.2) and “*malnutrition assessment standard based on the GLIM criteria”* (1.5.2). We further prioritized the “*configuration of consultation room environment, layout, and basic facilities”* (1.3.1), mandating validated assessment tools (e.g., body composition analyzers, handgrip dynamometers, food models). This addresses the gap identified by Moick et al. (20), who found that structural indicators were cited far less frequently than process indicators among 822 existing QIs.

**Process level:** Indicators such as “*standardized implementation rate of enteral and parenteral nutrition”* (2.2.1), “*standardized rate of patient nutritional risk screening and assessment”* (2.1.2), and “*completeness rate of outpatient medical record documentation”* (2.4.2) concretely operationalize the core workflow. Moick et al. (20) identified that among 822 nutrition care QIs extracted from 46 studies, documentation-related sub-themes were relatively under-represented among the 151 mapped sub-themes, highlighting a critical gap that our system addresses by incorporating explicit, formula-based documentation metrics (e.g., 2.4.2). Our system redresses these gaps by pairing each indicator with explicit calculation formulas, transforming vague requirements into auditable metrics.

**Outcome level:** We prioritized “*incidence of complications from nutrition support therapy”* (3.2.1) and “*improvement rate of patient quality of life”* (3.1.3). Moick et al. (20) reported that patient-reported outcome measures were addressed by merely 6% of 822 QIs—our inclusion of quality-of-life improvement directly responds to this gap.

Critical distinction from prior frameworks. Compared with generic outpatient frameworks (22, 23), which typically organize structural indicators around systems, environments, and human resources, and emphasize waiting time and generic health education in process indicators (23), our system delves into nutrition care's technical nuances. Unlike existing generic nursing quality tools (22), our indicators are tailored to the unique risk-benefit profile of NNCs. The recent development of practice standards for patient education in nurse-led clinics emphasizes that process standards guide NLC practice (24); our system advances this by pairing each nutrition-specific education process with quantifiable metrics. Furthermore, the weight distribution reflects a clear value orientation: the highest composite weight of the Structure dimension (0.637) emphasizes the importance of a solid foundation. Among specific indicators, “*emergency response plan for unexpected events”* (1.6.2), “*standardization of medical records and documentation”* (2.4.2), and “*improvement rate of patient quality of life”* (3.1.3) carry relatively high composite weights, clearly guiding clinical practice to prioritize patient safety and consistently target the improvement of patient health outcomes and quality of life as fundamental goals (25).

### 4.3 Feasibility and practicality of the indicator system

Operationalizability through quantifiable metrics. A critical weakness identified by Moick et al. (20) in their systematic review of 822 nutrition care QIs was the “poor operability” of existing indicators, largely due to vague documentation requirements and missing linkages between structure/process indicators and measurable outcomes. Our system directly addresses this gap by embedding explicit, formula-based evaluation criteria for the vast majority of its 61 tertiary indicators, as detailed in Supplementary Table S3. For example, “completeness rate of outpatient medical record documentation” (2.4.2) specifies the calculation formula: (Number of complete medical records/Number of records sampled) × 100%, with data sourced from medical record audits against documentation standards. Similarly, the “standardized rate of patient nutritional risk screening and assessment” (2.1.2) defines the numerator as “patients who received both screening and assessment using validated tools (e.g., NRS-2002, MUST)” and the denominator as “total eligible patients seen in the clinic.” These operationalized designs transform abstract quality concepts into auditable, low-subjectivity metrics—a decisive advancement over generic outpatient frameworks (23), which typically organize structural indicators around systems and environments but lack quantifiable formulas for process-level documentation.

Management leverage via the weighting system. Beyond individual metrics, the composite weight distribution provides nurse managers with a strategic prioritization tool. Prior research on outpatient nursing management quality (25) emphasizes that weighted indicators enable targeted resource allocation. In our system, the highest composite weights within the Process dimension cluster around assessment (2.1) and patient records and follow-up management (2.4), signaling that standardizing the technical core of nutrition care—including nutritional risk screening, medical record documentation, and remote follow-up—should be the immediate focus of quality improvement efforts. This aligns with Pouresmail et al.'s (24) recent development of practice standards for patient education in nurse-led clinics, which emphasized that “process standards guide practice in NLCs”—our system advances this by pairing each such standard with a quantifiable metric, enabling managers to track compliance objectively rather than relying on subjective impressions.

Application scenarios and future validation. The system supports three complementary applications: 1. internal self-assessment and continuous quality improvement (CQI) within individual NNCs, forming a closed loop of “evaluation-feedback-improvement”; 2. horizontal benchmarking across institutions under hospital administration; and 3. a reference framework for health authorities in formulating specialty accreditation standards. However, we acknowledge that the system has not yet undergone field testing in real clinical settings. While the quantifiable design reduces subjectivity in principle, its actual feasibility—including the time burden of data collection, inter-rater reliability of chart audits, and responsiveness to quality improvement interventions—requires empirical validation through prospective multi-center implementation studies.

### 4.4 Implications for nursing management

This theoretically informed and consensus-based index system provides nurse managers and administrators with a structured framework and a ready-to-use toolkit for enhancing quality in NNCs. By operationalizing the Donabedian model into specific, weighted indicators, it transforms abstract quality concepts into actionable management objectives. Managers can leverage this system to conduct systematic internal audits, identifying strengths and gaps across structure, process, and outcome dimensions. The assigned weights offer critical guidance for prioritization, enabling the strategic allocation of limited resources to high-impact areas such as staff competency development, safety protocol implementation, and patient outcome improvement. Furthermore, the system facilitates standardized benchmarking and continuous quality improvement (CQI) by establishing clear, measurable targets. It empowers leadership to make evidence-informed decisions, track progress over time, and demonstrate accountability in this specialized and evolving area of ambulatory care, ultimately supporting the goal of standardized, high-quality service delivery across different settings.

### 4.5 Limitations of the study

Despite the rigorous approach adopted in this study, several limitations warrant acknowledgment and should be addressed in future research. First, the study sample presents a selection bias regarding institutional tier. All 17 experts were recruited from domestic tertiary Grade A hospitals. While this ensured authoritative and cutting-edge perspectives, it necessitates caution regarding the generalizability of the derived indicator system to secondary hospitals and community health centers with more constrained resources. However, it is important to note that the expert panel embodied substantial geographic diversity, representing eight cities across eastern, southwestern, and northwestern China (Nanjing, Hangzhou, Zigong, Kunming, Changzhou, Zhangjiagang, Suzhou, and Ningxia). This geographic spread constitutes a methodological strength, ensuring that the indicator system reflects varied regional practices and mitigating urban-centric bias. Nevertheless, the inclusion of diverse geographic regions does not fully compensate for the lack of representation from primary and secondary care settings; therefore, the system's applicability across different institutional tiers requires further empirical validation. Variations in resource endowment and service delivery models at the primary care level may affect the relevance of certain indicators, such as those related to advanced equipment availability or multidisciplinary team establishment (26).

Second, the psychometric properties of the system require empirical validation. This study primarily focused on establishing the content validity and expert validity of the indicator system. However, other critical psychometric attributes—including construct validity (e.g., whether factor analysis confirms the hypothesized three-dimensional SPO structure), criterion-related validity (e.g., correlation between system scores and actual patient outcomes), and reliability (e.g., internal consistency and test-retest reliability)—have not yet been evaluated through large-scale, multi-center empirical studies (27). Third, the ratio of indicators to experts warrants consideration. Although the sample size of 17 experts is consistent with recommendations for Delphi studies, it is relatively small for the development of a comprehensive system containing 61 third-level indicators. Ideally, a larger expert panel would be preferable to validate such an extensive item pool. However, it is important to note that our panel consisted exclusively of senior professionals with high authority coefficients (Cr = 0.90 and 0.89). Moreover, the high degree of coordination among experts (significant Kendall's W, *P* < 0.01) suggests that despite the smaller number, the consensus reached was robust and reliable. Nevertheless, the potential influence of the expert-to-indicator ratio on the generalizability of the weights cannot be entirely dismissed. Future multi-center studies involving a broader range of clinicians are essential to empirically validate and potentially refine this system.

Fourth, the system must evolve to remain relevant. The fields of medical technology, nursing philosophy, and healthcare policy are in a state of rapid flux. Influences such as “Internet + Nursing Services” and reforms in DRG/DIP payment models are exerting a growing impact on outpatient clinic operations. Although the current indicator system touches upon related concepts, a dynamic updating mechanism is essential for the future. This mechanism should involve the periodic review and revision of both the indicators and their assigned weights in light of emerging policies, new evidence, and evolving clinical practices to ensure the system's continued contemporaneity and relevance (22).

### 4.6 Conclusion and future perspectives

This study successfully developed a preliminary, consensus-based quality evaluation index system for clinical NNCs, grounded in the Donabedian SPO model and informed by a two-round Delphi expert consultation. While the system provides a structured framework for assessing NNC quality, it should be recognized as an initial, theoretically derived tool that requires further methodological and clinical validation before widespread implementation. It represents a foundational step toward addressing the current gap in standardized evaluation tools for this specialty and offers a reference for promoting the high-quality and homogeneous development of NNCs in China. Future research should be dedicated to: Conducting multi-center empirical studies to validate and optimize the psychometric properties of this system; Developing supporting informatized evaluation tools or platforms to enhance the efficiency of data collection and analysis; Conducting interventional research based on this system to explore and validate best practice models for improving the quality of NNCs.

### 4.7 Adherence to reporting guidelines

This Delphi study followed the Reporting Checklist for Delphi Technique in Clinical Research Papers (26 items) developed by Liu et al. (28), which is consistent in scope and rigor with the CREDES guideline. The completed checklist is provided as Supplementary Table S4, with each item cross-referenced to the relevant page(s) in the manuscript.

## Data Availability

The raw data supporting the conclusions of this article will be made available by the authors, without undue reservation.
