# Supplementary material for: Construction of a quality evaluation index system for nutrition nursing clinics based on the Donabedian model
Source: Front Nutr. 2026 Sep 17;13:1867437. doi: 10.3389/fnut.2026.1867437 (PMC13627057; doi:10.3389/fnut.2026.1867437)
Supplement: Supplementary file 1 [file Table_1.docx]

Tables S1 . Details of Indicator Revisions after the First Round of Delphi Consultation

| **Revision Type** | **Revised Position (Round 2)** | **Corresponding Round 1 Entry/Position** | **Revision** | **Reason for Revision** | **Research Team's Adoption and Deepening Logic** |
| --- | --- | --- | --- | --- | --- |
| Secondary Indicator Split | 1.4 System 1.5 Internet+ Related System 1.6 Work Standard 1.7 Standardized Process | 1.4 System Duties and Responsibilities | Split into 4 independent secondary indicators. | Experts 7, 15: "Separate system, work standards, and duties/responsibilities." | The opinion was adopted. The original "system duties and responsibilities" was deemed too broad. Splitting it into the four dimensions of "System", "Internet+ System", "Work Standard", and "Standardized Process" allows for a more systematic and refined assessment of the clinic's structured development at different levels (top-level design, digital norms, technical standards, operational processes), aligning with modern quality management theory. |
| Secondary Indicator Addition | 3.6 Team Collaboration | Addition | Added this secondary indicator under the Outcome dimension. | Expert 1: "Is it necessary to add multi-disciplinary collaboration?" | The opinion was adopted. It was recognized that the effectiveness of multi-disciplinary collaboration (e.g., referrals, consultations, information sharing) is a direct reflection of the clinic's integrated care capability, regional influence, and operational efficiency, and thus should be evaluated as an important "outcome". This dimension was added to comprehensively assess external collaboration outcomes. |
| Tertiary Indicator Deletion | (None) | 3.1.2 Incidence of acute/chronic complications of malnutrition and sarcopenia  1.4.7 Emergency response plan for outpatients with common conditions including malnutrition and sarcopenia | Deleted. | 3.1.2: Expert 11: "Most patients visit the nutrition clinic for malnutrition; the outcome should be the improvement rate of nutritional status. This item can be deleted."  1.4.7: Failed to meet the pre-specified retention criterion: coefficient of variation (CV) = 0.284, exceeding the threshold of <0.25. Importance score = 4.18 (met the mean ≥3.5 criterion). | 3.1.2: The opinion was adopted. Further analysis concluded that this indicator had a logical misplacement: "Complication incidence" is inherently a measure of safety and negative events, more suited to the "Safety (Adverse) Events" category. The design intent of the "Patient Outcomes" dimension is to measure positive health improvements. Its deletion makes the focus of "3.1 Patient Outcomes" purer and more positive.  1.4.7: Following team deliberation, the indicator was removed because its excessively high CV indicated low expert consensus on this item. Although its importance score met the threshold, the research team determined that indicators failing the CV criterion should be excluded to ensure the robustness and reliability of the final indicator. |
| Tertiary Indicator Addition (Based on Explicit Suggestion) | 1.1.5 Nurse Communication Skills Level 1.1.6 Nurse Health Education Skills Level | Addition | Added these two soft skills indicators under "Human Resources". | Experts 7, 11, 17: "1. Position division (setup); 2. Communication and health education skills level." | The opinion was adopted. It was recognized that for nutrition nursing clinics, which are centered on consultation, education, and behavioral intervention, nurses' communication and health education skills are as crucial as professional technical knowledge. They are key "soft skills" directly impacting patient understanding, trust, compliance, and final intervention outcomes, and must be emphasized and assessed separately within core personnel competencies. |
|  | 2.1.7 Patient and Family Compliance Assessment | Addition | Added this indicator under "Assessment and Diagnosis". | Expert 6: "Patient and family compliance assessment." | The opinion was adopted. It was recognized that systematically assessing patients' and their families' level of understanding, support system, willingness to implement, and potential barriers during the initial assessment phase constitutes the fundamental prerequisite for subsequently formulating individualized and actionable nutrition intervention plans. The addition of this indicator strengthens the foresight and comprehensiveness of the assessment process. |
| Tertiary Indicator Wording and Quantification Optimization | 1.1.3 Nurse Core Competency Assessment Pass Rate | 1.1.3 Nurse Professional Ability Performance | Changed to a quantitative expression, specifying the assessment method (OSCE case simulation) and pass standard (≥85 points). | Expert 16: "Suggest changing to 'Nurse Core Competency Assessment Pass Rate', easier to quantify. Suggest clarifying the pass standard, e.g., a score ≥85 points indicates a pass." | The opinion was adopted. The original "professional ability performance" was vague and not objectively measurable. The revision achieved a fundamental shift from qualitative description to quantitative assessment: "Pass rate" is a clear output metric. Combined with the specified assessment method and pass threshold, the indicator became measurable, comparable, and assessable, greatly enhancing the tool's operability. |
| Tertiary Indicator Position Adjustment | 3.5.6 Research and Innovation Achievements | 1.1.5 Research and Innovation Achievements | Moved from "Structure - Human Resources" to "Outcome - Sustainable Development Indicators". | Experts 1, 17 mentioned "clinical specialty training experience" in the discussion of the original item, leading the research team to conclude the focus was on qualifications rather than the achievements themselves. | Logical reclassification based on indicator intrinsic attributes. Although no expert directly requested the move, the team analyzed that "Research and Innovation Achievements" represent the academic output and development potential generated by nurses or the team after possessing good capability (structure). It is a manifestation of the clinic's academic influence, innovation capability, and long-term development level, belonging to the "development" dimension within "Outcome Quality". This adjustment clarifies the logical chain of the SPO model (Structure → Process → Outcome). |
|  | 3.5.7 Clinical Service Coverage/Influence | 3.4.6 Clinical Service Coverage/Influence | Moved from "Health Efficiency and Benefit Indicators" to "Sustainable Development Indicators". | Expert 16 (explicit suggestion): "Suggest moving this item to 3.5 Sustainable Development Indicators." | The opinion was directly adopted and fully endorsed. "Clinical Service Coverage/Influence" (e.g., proportion of out-of-town patients, referral rate from lower-level hospitals) reflects the clinic's brand reputation, regional competitiveness, and long-term development potential, which differs in nature from short-term, direct economic benefit indicators like "per capita treatment cost" or "cost control". Placing it under "Sustainable Development Indicators" allows for a more accurate measurement of its long-term impact and market expansion capability. |
| Comprehensive Upgrade of Tertiary Indicator Evaluation Details | The "Evaluation Details" for all tertiary indicators | The "Evaluation Method" column from Round 1 indicators | Developed detailed, quantifiable evaluation details for each indicator, including calculation formulas, data sources, measurement tools, and evaluation thresholds. | Systematic response to frequent, specific opinions from multiple experts, for example: • Expert 16 (on 3.1.3): "Need to clearly define the standard for 'improvement', which nutritional indicators..." • Experts 2, 16 (on 2.1.3): "Suggest specifying which standardized assessment tool or questionnaire to use." • Expert 4 (on 2.4.1): Proposed a specific calculation formula. • Expert 10 (on 1.3.7): "Suggest adding 'appropriateness' to the evaluation method." | The research team recognized that the overly vague "Evaluation Method" in the first round was the core pain point preventing the indicator system from being implemented. Therefore, synthesizing all expert opinions advocating for "clarification", "tool specification", and "formula provision", a revolutionary revision was made to the assessment method for every tertiary indicator. This fundamentally transformed the system from a "theoretical framework" into a "practical tool" that hospitals can immediately execute and audit, ensuring the final research output possesses genuine practical value. |
