# Supplementary material for: Construction of a quality evaluation index system for nutrition nursing clinics based on the Donabedian model
Source: Front Nutr. 2026 Sep 17;13:1867437. doi: 10.3389/fnut.2026.1867437 (PMC13627057; doi:10.3389/fnut.2026.1867437)
Supplement: Supplementary file 2 [file Table_2.docx]

Tables S2. Details of Indicator Revisions after the Second Round of Delphi Consultation

| **Revision Type** | **Revised Position (Final Version)** | **Corresponding Round 2 Entry/Position** | **Revision Content** | **Direct Reason for Revision (Based on Expert Opinion)** | **Research Team's Adoption and Deepening Logic** |
| --- | --- | --- | --- | --- | --- |
| Secondary Indicators: Restructuring and Consolidation | Structural Adjustment: Final secondary indicators consolidated from 17 to 16. | Original 1.4 System, 1.5 Internet+ System, 1.6 Work Standard, 1.7 Standardized Process, etc. | The 7 secondary indicators (1.1-1.7) under the Structure dimension were integrated and re-categorized into 6: 1.1 Human Resources, 1.2 Personnel Assessment & Training, 1.3 Environment & Facilities, 1.4 System, 1.5 Work Standard, 1.6 Standardized Process. | Expert 16 suggested merging "1.2 Personnel Training" with "1.1 Human Resources". Experts 2, 15 suggested merging "1.5 Internet+ Related System" with "1.4 System". Expert 16 pointed out that "1.6 Work Standard" and "1.7 Standardized Process" are conceptually overlapping. | The opinions were adopted. The research team recognized the need for a more streamlined and logical structure. A significant integration and restructuring were performed, moving from 7 to 6 secondary indicators within the Structure dimension to reduce redundancy and improve clarity, though a complete merger into "SOP" was not implemented. |
| Secondary Indicators: Position Adjustment and Addition | New Secondary Indicators: 2.5 Nutrition Nursing Clinic Quality Improvement; 2.6 Collaboration. | (Content from original 3.5 Sustainable Development Indicators, 3.6 Team Collaboration) | Two new secondary indicators were added under "Process Indicators", moving development and collaboration-related items from the "Outcome" dimension. | Expert 16 indicated that "Sustainable Development" belongs to organizational development indicators and should not be a routine 'Outcome Indicator'". Experts 4, 16 pointed out that "3.6 Team Collaboration" is a process quality indicator and was misclassified. | The opinions on the fundamental attributes of indicators were adopted. The team agreed that "quality improvement" activities and "collaboration" are core components of the care *process*. Reclassifying them under the Process dimension aligns strictly with Donabedian's theory and corrects a logical error in the preliminary framework. |
| Tertiary Indicators: Extensive Deletion and Consolidation | Significant streamlining of Structure dimension indicators. | Original 1.1.1, 1.1.5, 1.1.6, 1.4.1-1.4.12, 1.5.1-1.5.5, and many others. | Deleted or consolidated 46 tertiary indicators. (e.g., Deleted "1.1.1 Nurse composition...", "1.1.5/6 Communication & Health Ed."; Consolidated many system items into core ones like "1.4.1 HAIS Management System"). | Multiple experts (1, 3, 6, 11, 15, 16, etc.) provided feedback on "focusing on the core, avoiding redundancy, improving operability", questioning vague or overlapping indicators. | A decisive shift from "comprehensive listing" to "focused core" was made. The team rigorously applied the principles of parsimony and operability, deleting indicators deemed non-essential, consolidating duplicate entries, and highly streamlining the system to enhance its practical utility and reduce assessment burden. |
| Tertiary Indicators: Wording and Evaluation Detail Optimization | 1.2.4 Rate of Nurses Meeting Training Frequency Standards | Original 1.2.6 Rate of Nurses Meeting Training Frequency Standards | Evaluation details optimized from vague frequencies to: "Basic theory & knowledge at least every 6 months; Annual specialized skill retraining completion rate 100%, new knowledge training ≥2 times/year." | Expert 16: "Is 'once every six months'/'once a year' the minimum or the target?" Suggested the specific quantified standard. | The opinion was directly adopted. The ambiguous phrasing was replaced with a clear, measurable, and actionable standard, transforming the indicator from a descriptive suggestion to a quantifiable performance metric. |
|  | 2.1.4 Patient and Family Compliance Assessment | Original 2.1.7 Patient and Family Compliance Assessment | Evaluation method changed from a simplistic formula to: "Comprehensive judgment using patient diaries, follow-up statements, dispensing records, or standardized scales (e.g., Morisky)." | Expert 16: Pointed out the original formula was "difficult to measure precisely in nutrition support" and provided the specific improvement suggestion. | The opinion was directly adopted. The team recognized the impracticality of the original formula for nutrition adherence. The revised method offers flexible, feasible, and clinically relevant approaches to assess a complex construct like compliance. |
|  | 3.4.1 Total Cost of Nutrition Nursing Care | Original 3.4.1 Total Cost of Nutrition Nursing Care | Evaluation detail changed from "actual per capita cost" to "average cost per visit controlled within regional DRG/DIP payment standards." | Expert 16: Pointed out that "actual per capita cost" has no reference frame and is meaningless alone. Suggested using medical insurance payment standards as a benchmark. | The opinion was directly adopted. The standalone cost figure was replaced with a context-rich, policy-relevant benchmark (DRG/DIP), making the indicator meaningful for both cost control and reimbursement alignment. |
| Tertiary Indicators: Position Adjustment | 2.6.1 Implementation of Multidisciplinary Team Work 2.6.3 Standardized Rate of Two-Way Referral | Original 3.6.5 Management of Multidisciplinary Team Original 3.6.1 Volume of Two-Way Referral | Moved from "Outcome - Team Collaboration" to "Process - Collaboration". Changed from "volume" to "quality process" indicators (e.g., Standardized Rate). | Experts 4, 16: Pointed out that "Team Collaboration" is a process, not an outcome. Expert 16: Pointed out that "Volume of Two-Way Referral" is a quantity record, not reflecting quality, and suggested "Standardized Implementation Rate". | The opinions were adopted. This adjustment corrects the classification of collaboration activities. Furthermore, shifting the focus from mere "volume" to "quality of process" (e.g., standardized rate) aligns with the core purpose of a *quality*evaluation system. |
