# Supplementary material for: Construction of a quality evaluation index system for nutrition nursing clinics based on the Donabedian model
Source: Front Nutr. 2026 Sep 17;13:1867437. doi: 10.3389/fnut.2026.1867437 (PMC13627057; doi:10.3389/fnut.2026.1867437)
Supplement: Supplementary file 3 [file Table_3.docx]

Table S3**. Clinical Nutrition Nursing Clinic Quality Evaluation Indicator System**

| Table 4. Clinical Nutrition Nursing Clinic Quality Evaluation Indicator System | | | | | |
| --- | --- | --- | --- | --- | --- |
| Indicator | Evaluation Details | Importance (Score) Mean ± SD | Coefficient of Variation | Local Weight | Composite Weight |
| 1 Structure |  | 4.765±0.437 | 0.0918 | 0.637 | - |
| 1.1 Human Resources |  | 4.882±0.332 | 0.068 | 0.327 | 0.2083 |
| 1.1.1 Composition Ratio of Nurses' Education Level, Professional Title, Competency Level, and Provincial or Higher-Level Specialist Nurses | Review personnel files to calculate the composition ratio. | 4.941±0.243 | 0.0491 | 0.4396 | 0.0915 |
| 1.1.2 Qualification Requirements for Nutrition Nursing Clinic Staff | Requirements: bachelor's degree or above in nursing; National Registered Nurse License; provincial-level specialist nurse certification or clinical specialty training experience; mid-level title (Charge Nurse) or above, or ≥5 years of clinical nutrition nursing experience. | 4.882±0.332 | 0.068 | 0.3331 | 0.0694 |
| 1.1.3 Staffing Plan for the Nutrition Nursing Clinic | The hospital has a clear staffing plan. | 4.000±0.866 | 0.2165 | 0.1056 | 0.022 |
| 1.1.4 Employment Status of Nurses (Full-time, Part-time) | Calculate the proportion of full-time nurses: (Number of full-time nurses / Total number of nurses) × 100%. | 3.941±0.748 | 0.1897 | 0.0738 | 0.0154 |
| 1.1.5 Establishment of a Multidisciplinary Team (including Dietitian/Physician/Pharmacist/Nurse/Rehabilitation Therapist) | Calculate the ratio of (dietitians + physicians + nurses + pharmacists) to outpatient visits served. | 3.706±0.849 | 0.2291 | 0.0479 | 0.01 |
| 1.2 Personnel Assessment and Training |  | 3.765±0.752 | 0.1999 | 0.069 | 0.0439 |
| 1.2.1 Coverage Rate of Nutrition Specialty Nursing Training Content | Core content coverage is 100%. Review the training syllabus and records. | 4.000±0.791 | 0.1976 | 0.1056 | 0.0046 |
| 1.2.2 Pass Rate of Nutrition Specialty Nursing Theory Training Assessment | Check assessment score reports. Pass standard: score ≥80; pass rate ≥90%. | 4.882±0.332 | 0.068 | 0.4396 | 0.0193 |
| 1.2.3 Pass Rate of Nutrition Nursing Operation Skills Assessment | Check assessment score reports. Pass standard: score ≥90; pass rate ≥90%. | 3.647±0.606 | 0.1663 | 0.0479 | 0.0021 |
| 1.2.4 Rate of Nurses Meeting Training Frequency Standards | Basic theory and professional knowledge at least once every six months; annual specialized skill retraining completion rate of 100%; new knowledge training ≥2 times/year. | 3.882±0.781 | 0.2012 | 0.0738 | 0.0032 |
| 1.2.5 Pass Rate of Nurse Core Competency Assessment | Core competencies are based on national or industry-published clinical nutrition nursing core competency standards. Pass standard: score ≥85; pass rate ≥90%. | 4.765±0.437 | 0.0918 | 0.3331 | 0.0146 |
| 1.3 Environment and Facilities |  | 4.941±0.243 | 0.0491 | 0.4119 | 0.2624 |
| 1.3.1 Configuration of Consultation Room Environment, Layout, and Basic Facilities | Functional areas are clearly divided (waiting area, assessment area, education area). Check the compliance rate against a standard configuration checklist (e.g., height-weight scale, body composition analyzer, skinfold caliper, soft tape measure, handgrip dynamometer, blood pressure monitor, blood glucose meter, food models, food pagoda model, resting energy measurement device). | 4.882±0.332 | 0.068 | 0.5363 | 0.1407 |
| 1.3.2 Functional Integrity Rate of Nutrition Equipment | Check equipment maintenance records. Calculation: (Number of normally functioning devices / Total number of devices) × 100%. | 3.706±0.686 | 0.1851 | 0.0722 | 0.0189 |
| 1.3.3 Completeness of Nutrition-related Medications, Materials, and Emergency Supplies | Simulate an emergency scenario to check item availability (response time ≤3 minutes). The qualification rate for medication and instrument expiration dates is 100%. | 4.176±0.883 | 0.2114 | 0.1996 | 0.0524 |
| 1.3.4 Accessibility and Appropriateness of Health Education Materials | Patients evaluate the convenience of accessing health education materials. Materials are reviewed by an expert panel for comprehensiveness, scientific content, and appropriate format. | 4.118±0.781 | 0.1897 | 0.1447 | 0.038 |
| 1.3.5 Electronic Medical Records, Nutrition Management Software, and Follow-up Informatization Development | Rate of informatized records = (Number of informatized records / Total number of records) × 100%. | 3.529±0.717 | 0.2033 | 0.0473 | 0.0124 |
| 1.4 System |  | 3.588±0.795 | 0.2216 | 0.0386 | 0.0246 |
| 1.4.1 Healthcare-Associated Infection Management System for the Nutrition Nursing Specialty Clinic | System evaluation: 0.5 × (Completeness rate of core system documents) + 0.5 × (Employee awareness rate). | 4.941±0.243 | 0.0491 | 0.5 | 0.0123 |
| 1.4.2 Clear Remuneration and Performance Appraisal System for Nurses in the Nutrition Nursing Specialty Clinic | Develop a scientific, quantifiable remuneration and performance appraisal plan with a clear structure (base salary, performance bonus, special allowances) and detailed evaluation criteria; survey nurses' level of acceptance of the plan. | 4.941±0.243 | 0.0491 | 0.5 | 0.0123 |
| 1.5 Work Standards |  | 3.941±0.899 | 0.2282 | 0.0988 | 0.0629 |
| 1.5.1 Nutritional Risk Screening Standard | Use tools such as NRS-2002, MUST, MST. Standard evaluation: 0.5 × (Completeness rate of core work standards) + 0.5 × (Employee mastery rate). | 3.824±0.728 | 0.1903 | 0.0453 | 0.0028 |
| 1.5.2 Malnutrition Assessment Standard | Develop malnutrition assessment standards based on guidelines and consensus, such as the GLIM criteria. Standard evaluation: 0.5 × (Completeness rate of work standards) + 0.5 × (Employee execution rate). | 3.824±0.809 | 0.2116 | 0.0453 | 0.0028 |
| 1.5.3 Nutrition Intervention Standard | Based on the five-step treatment model, specify indications, dosage, and monitoring indicators for EN/PN, oral supplements, and dietary prescriptions. Standard evaluation: 0.5 × (Completeness rate of work standards) + 0.5 × (Employee mastery rate). | 4.941±0.243 | 0.0491 | 0.3421 | 0.0215 |
| 1.5.4 Standard for Hospital Admission due to Malnutrition | Develop relevant standards based on guidelines, consensus, and clinical experience. Patients meeting any one or a combination of the specified conditions should be determined as requiring inpatient treatment for malnutrition. Standard evaluation: 0.5 × (Completeness rate of work standards) + 0.5 × (Employee execution rate). | 4.529±0.717 | 0.1584 | 0.1613 | 0.0102 |
| 1.5.5 Health Education Standard | Provide written/electronic Nutrition Guidance Manual and other forms of health education methods. Evaluation: 0.5 × (Completeness rate of work standards) + 0.5 × (Employee execution rate). | 3.882±0.781 | 0.2012 | 0.064 | 0.004 |
| 1.5.6 Follow-up and Monitoring Standard | Determine follow-up frequency, monitoring indicators (anthropometric, body composition, laboratory biochemistry, physical function, quality of life, etc.)), and the timing and documentation requirements for subjective assessments (e.g., NRS-2002, MNA-SF, PG-SGA) and efficacy evaluations. Standard evaluation: 0.5 × (Completeness rate of core work standards) + 0.5 × (Employee mastery rate). | 4.941±0.243 | 0.0491 | 0.3421 | 0.0215 |
| 1.6 Standardized Processes |  | 3.706±0.849 | 0.2291 | 0.0548 | 0.0349 |
| 1.6.1 Standardized Process for Nursing Technical Operations | E.g., processes for oral nutritional supplementation, tube placement, enteral nutrition infusion, percutaneous endoscopic gastrostomy/jejunostomy tube care. Process evaluation: 0.5 × (Completeness rate of core processes) + 0.5 × (Employee mastery rate). | 3.824±0.883 | 0.2309 | 0.057 | 0.002 |
| 1.6.2 Emergency Response Plan Process for Unexpected Events | The emergency plan comprehensively covers risks specific to the nutrition nursing clinic (e.g., aspiration, allergic reaction, hypoglycemia, falls, tube dislodgement, clogging), and there are drill records. | 4.882±0.332 | 0.068 | 0.4414 | 0.0154 |
| 1.6.3 Standardization of Medical Records and Documentation | Complete documentation of dual diagnosis (primary disease diagnosis + nutrition diagnosis) and the three-level diagnosis of malnutrition (nutrition screening - nutrition assessment - comprehensive evaluation, dietary prescription, intervention measures, follow-up plan). Document evaluation: 0.5 × (Completeness rate of core standardized documents) + 0.5 × (Employee awareness rate). | 4.824±0.529 | 0.1096 | 0.2508 | 0.0087 |
| 1.6.4 Collaboration and Division of Labor Process between the Nutrition Nursing Clinic and the Nutrition Medical Clinic | (Detailed process description omitted). Process evaluation: 0.5 × (Completeness rate of core process documents) + 0.5 × (Employee execution rate). | 4.824±0.393 | 0.0815 | 0.2508 | 0.0087 |
| 2 Process Indicators |  | 4.000±0.866 | 0.2165 | 0.1047 | - |
| 2.1 Assessment |  | 4.941±0.243 | 0.0491 | 0.2532 | 0.0265 |
| 2.1.1 Completeness of General Information Assessment | Review sampled cases. Completeness rate = (Number of cases with complete general information / Total number of cases sampled) × 100%. | 4.941±0.243 | 0.0491 | 0.4107 | 0.0109 |
| 2.1.2 Standardized Rate of Patient Nutritional Risk Screening and Assessment | Standardized rate = (Number of cases using standardized assessment/diagnostic methods / Number of cases sampled) × 100%. | 3.588±0.795 | 0.2216 | 0.0451 | 0.0012 |
| 2.1.3 Assessment of Patient Nutrition Literacy and Self-Management Ability | Use standardized assessment tools or questionnaires for evaluation, such as the Comprehensive Health Literacy Instrument for Nutrition (CHI-NLit) and the Nutrition Self-efficacy Questionnaire (NSEQ). | 4.412±0.712 | 0.1615 | 0.1335 | 0.0035 |
| 2.1.4 Assessment of Patient and Family Compliance | Make a comprehensive judgment based on patient diaries, follow-up statements, or pharmacy/nutrition preparation dispensing records, or use adherence scales like Morisky during follow-up for assessment. | 4.941±0.243 | 0.0491 | 0.4107 | 0.0109 |
| 2.2 Nutrition Nursing Intervention |  | 4.765±0.437 | 0.0918 | 0.1537 | 0.0161 |
| 2.2.1 Standardized Implementation Rate of Enteral and Parenteral Nutrition | Standardized implementation rate = (Number of execution records meeting requirements / Total number of execution records) × 100%. | 4.824±0.393 | 0.0815 | 1 | 0.0161 |
| 2.3 Health Education |  | 4.059±0.899 | 0.2216 | 0.0545 | 0.0057 |
| 2.3.1 Education on Knowledge and Skills Related to Oral Nutrition, Enteral Nutrition, Parenteral Nutrition, and Diet | Review health education records in sampled medical records; calculate the completion rate of education content. | 3.529±0.800 | 0.2266 | 0.0425 | 0.0002 |
| 2.3.2 Education on Knowledge and Skills Related to Nutrition Therapy Combined with Exercise Therapy | (Details as per the description of the evaluation target). | 3.765±0.664 | 0.1764 | 0.0648 | 0.0004 |
| 2.3.3 Education on Self-Monitoring Indicators for Malnutrition/Sarcopenia (e.g., weight, calf circumference, walking speed) | (Details as per the description of the evaluation target). | 4.941±0.243 | 0.0491 | 0.4603 | 0.0026 |
| 2.3.4 Education on Prevention and Management of Malnutrition/Sarcopenia and Its Complications | (Details as per the description of the evaluation target). | 3.882±0.928 | 0.2389 | 0.0961 | 0.0005 |
| 2.3.5 Education on Healthy Lifestyles such as Smoking Cessation, Alcohol Limitation, and Maintaining Weight/Muscle Mass | (Details as per the description of the evaluation target). | 4.882±0.332 | 0.068 | 0.3363 | 0.0019 |
| 2.4 Patient Records and Follow-up Management |  | 4.941±0.243 | 0.0491 | 0.2532 | 0.0265 |
| 2.4.1 Patient Record Filing Rate | Filing rate = (Number of patients with filed records / Total number of patients seen) × 100%. | 4.118±0.781 | 0.1897 | 0.0988 | 0.0026 |
| 2.4.2 Completeness Rate of Outpatient Medical Record Documentation | Documentation completeness rate = (Number of complete medical records / Number of records sampled) × 100% (checked item by item against documentation standards). | 4.882±0.332 | 0.068 | 0.3258 | 0.0086 |
| 2.4.3 Timeliness of Remote Follow-up | Timely remote follow-up rate = (Number of cases followed up on schedule / Number of cases requiring follow-up) × 100% (allowable error ≤3 days). | 3.882±0.857 | 0.2209 | 0.0547 | 0.0015 |
| 2.4.4 Patient Remote Follow-up Rate | Remote follow-up completion rate = (Number of patients who completed follow-up / Total number of patients requiring follow-up) × 100%. | 4.941±0.243 | 0.0491 | 0.4459 | 0.0118 |
| 2.4.5 Completeness and Accuracy of Record and Follow-up Information | Information accuracy rate = (Number of records with error-free information / Number of records sampled) × 100%. | 3.941±0.899 | 0.2282 | 0.0749 | 0.002 |
| 2.5 Nutrition Nursing Clinic Quality Improvement |  | 4.941±0.243 | 0.0491 | 0.2532 | 0.0265 |
| 2.5.1 Quality Improvement Project Completion Rate | Improvement completion rate = (Number of projects completed as planned / Total number of annual planned projects) × 100%. | 4.941±0.243 | 0.0491 | 1 | 0.0265 |
| 2.6 Collaboration |  | 3.706±0.849 | 0.2291 | 0.0323 | 0.0034 |
| 2.6.1 Implementation of Multidisciplinary Team/Nutrition Support Team Work | The hospital's multidisciplinary team includes members from the nutrition nursing clinic, and there are verifiable actual work records. | 4.118±0.857 | 0.2083 | 0.1995 | 0.0007 |
| 2.6.2 Inter-departmental Information Sharing Rate | Proportion of cases where nutritional assessments and treatment plans are synchronously updated in the EMR. | 3.706±0.772 | 0.2083 | 0.0703 | 0.0002 |
| 2.6.3 Standardized Rate of Two-Way Referral | Review actual records of two-way referrals of patients between higher-level hospitals, lower-level hospitals, and the hospital's own nutrition clinic. | 3.765±0.831 | 0.2208 | 0.0994 | 0.0003 |
| 2.6.4 Timeliness Rate of Consultation Response and Execution Rate of Consultation Opinions | Review medical records. | 4.941±0.243 | 0.0491 | 0.6308 | 0.0021 |
| 3 Outcome Indicators |  | 4.294±0.920 | 0.2141 | 0.2583 | - |
| 3.1 Patient Outcomes |  | 4.118±0.857 | 0.2083 | 0.1143 | 0.0295 |
| 3.1.1 Qualified Rate of Patient Mastery of Nutrition Self-Management Knowledge and Skills | Score patients via questionnaire survey. | 4.588±0.618 | 0.1348 | 0.3445 | 0.0102 |
| 3.1.2 Improvement Rate of Patient Nutritional Indicators | (Detail description omitted). Nutritional indicator improvement rate = (Number of patients with improved nutritional indicators per year / Total number of annual visits) × 100%. | 3.941±0.827 | 0.2098 | 0.1085 | 0.0032 |
| 3.1.3 Improvement Rate of Patient Quality of Life | It is recommended to use standardized quality of life assessment scales, such as SF-36 and QLQ-C30. Quality of life improvement rate = (Number of patients with improved quality of life scale scores per year / Total number of annual visits) × 100%. | 4.647±0.702 | 0.151 | 0.5469 | 0.0162 |
| 3.2 Occurrence of Safety (Adverse) Events |  | 4.824±0.393 | 0.0815 | 0.4067 | 0.105 |
| 3.2.1 Incidence of Complications from Nutrition Support Therapy | Complication rate = (Number of patients with complications / Total number of patients receiving nutrition support) × 100%. | 3.824±0.883 | 0.2309 | 0.1947 | 0.0204 |
| 3.2.2 Incidence of Nutrition-Related Hospital-Acquired Infections | Infection incidence rate = (Number of patients with hospital-acquired infections / Total number of all patients) × 100%. | 4.647±0.702 | 0.151 | 0.7172 | 0.0753 |
| 3.2.3 Adverse Event Incidence Rate | Adverse event incidence rate = (Number of adverse event incidents / Total number of procedures) × 1000‰. | 3.471±0.717 | 0.2067 | 0.0881 | 0.0093 |
| 3.3 Satisfaction |  | 3.882±0.857 | 0.2209 | 0.0723 | 0.0187 |
| 3.3.1 Satisfaction of Patients and Families with the Nutrition Nursing Clinic (including third-party) | Satisfaction scores from patients and families. | 4.176±0.883 | 0.2114 | 0.1267 | 0.0024 |
| 3.3.2 Nurse Satisfaction with Work in the Nutrition Nursing Clinic | Satisfaction scores from nurses. | 4.882±0.332 | 0.068 | 0.4901 | 0.0092 |
| 3.3.3 Satisfaction of Collaborating Physicians | Satisfaction scores from physicians, including clinical doctors and nutrition department doctors. | 3.588±0.712 | 0.1985 | 0.0498 | 0.0009 |
| 3.3.4 Complaint Incidence Rate | Complaint incidence rate = (Number of complaint incidents during the specified statistical period / Total number of patients during the specified statistical period) × 100%. | 4.824±0.393 | 0.0815 | 0.3335 | 0.0062 |
| 3.4 Health Efficiency and Benefit Indicators |  | 4.824±0.393 | 0.0815 | 0.4067 | 0.105 |
| 3.4.1 Total Cost of Nutrition Nursing Care | Average cost per visit is controlled within the regional DRG/DIP disease payment standard range. | 3.941±0.899 | 0.2282 | 0.1471 | 0.0154 |
| 3.4.2 Re-visit Rate for the Same Condition after Patient Recovery | Re-visit rate for the same condition = (Number of patients re-visiting for the same condition / Total number of recovered patients) × 100%. | 3.941±0.827 | 0.2098 | 0.1471 | 0.0154 |
| 3.4.3 Patient Follow-up Visit Rate | Follow-up visit rate = (Number of patients attending scheduled follow-up visits / Total number of patients requiring follow-up) × 100%. | 3.882±0.857 | 0.2209 | 0.0875 | 0.0092 |
| 3.4.4 Nutrition-Related Unplanned Hospitalization Rate | Nutrition-related unplanned hospitalization rate = (Number of patients hospitalized due to malnutrition or complications within 30 days post-intervention / Total number of nutrition clinic patients) × 100%. | 4.882±0.332 | 0.068 | 0.6184 | 0.065 |
