# Supplementary material for: Construction of a quality evaluation index system for nutrition nursing clinics based on the Donabedian model
Source: Front Nutr. 2026 Sep 17;13:1867437. doi: 10.3389/fnut.2026.1867437 (PMC13627057; doi:10.3389/fnut.2026.1867437)
Supplement: Supplementary file 4 [file Table_4.docx]

Supplementary Table S4. Reporting Checklist for Delphi Technique in Clinical Research Papers (26 Items)

| **Item No.** | **Checklist Category** | **Checklist Item**  **(Detailed Content)** | **Availability** | **Section in Manuscript** |
| --- | --- | --- | --- | --- |
| 1 | Title | Title reflects research topic and Delphi method | Yes | Title |
| 2 | Abstract | Structured summary reflecting complete Delphi process | Yes | Abstract |
| 3 | Authorship | Authors' professional/educational background and affiliations | Yes | Authorship |
| 4 | Funding | Funding source and grant number if applicable | Yes | Funding Declaration |
| 5 | Background & Objectives | Research background, significance, innovation, and objectives | Yes | Introduction |
| 6 | Methodology & Paradigm | Delphi appropriateness + research paradigm explanation | Yes | Introduction |
| 7 | Time Management | Study duration and interval between rounds | Yes | 1.3.3 Implementation of the Expert Consultation |
| 8 | Steering Group | Coordination group size, roles, and characteristics | Yes | 1.1 Establishment of the Research Team |
| 9 | Consensus Question List | Basis for forming question list (literature, evidence, empirical study) | Yes | 1.2 Literature Search and Development of the Initial Item Pool |
| 10a | Expert Questionnaire | Structured questionnaire with background, content, and open-ended questions | Yes | 1.3.1 Design of the Expert Consultation Questionnaire |
| 10b | Pilot Testing | Small-scale pre-test before formal consultation | No | — |
| 11a | Expert Recruitment | Recruitment method (purposive, criterion, snowball) | Yes | 1.3.2 Selection of Consulting Experts |
| 11b | Inclusion Criteria | Expert inclusion criteria (expertise and willingness) | Yes | 1.3.2 Selection of Consulting Experts |
| 11c | Panel Size & Types | Panel size and expert types (multidisciplinary) | Yes | 1.3.2 Selection of Consulting Experts; 2.1Demographic Characteristics of the Expert Panel |
| 11d | Delivery Method | Delivery method (email, WeChat, paper, etc.) | Yes | 1.3.3 Implementation of the Expert Consultation |
| 12 | Anonymity | Anonymity level, maintenance, and confirmation | Yes | 1.3.3 Implementation of the Expert Consultation |
| 13 | Iteration | Rounds, purpose of each round, transition plan | Yes | 1.3.3 Implementation of the Expert Consultation |
| 14 | Feedback | Feedback mode (qualitative/quantitative), method, and content | Yes | 1.3.3 Implementation of the Expert Consultation |
| 15 | Weights | AHP or other methods for priority ranking | Yes | 1.4.2 Analytic Hierarchy Process |
| 16 | Consensus Degree | Consensus coefficient | Yes | 1.4.1 Delphi Survey Analysis |
| 17a | Qualitative Analysis | Qualitative data analysis method and software | Yes | 1.4.1 Delphi Survey Analysis |
| 17b | Quantitative Analysis | Quantitative data analysis method and software | Yes | 1.4.1 Delphi Survey Analysis |
| 17c | Main Indicators | Response rate, authority, coordination, central tendency | Yes | 1.4.1 Delphi Survey Analysis |
| 17d | Other Indicators | AHP weights, consistency coefficients, etc. | Yes | 1.4.2 Analytic Hierarchy Process (AHP);  2.3 Degree of Coordination of Expert Opinions |
| 17e | Stopping Criteria | Pre-set stopping thresholds for Delphi | Yes | Methods (Delphi Consultation Process) |
| 18 | Ethics | Ethics committee approval and documentation | Yes | Ethics approval and consent to participate |
| 19 | Expert Information | Expert demographic characteristics | Yes | 2.1 Demographic Characteristics of the Expert Panel |
| 20a | Response Rate | Number of respondents per round and comments provided | Yes | 2.2 Level of Engagement and Authority of the Experts |
| 20b | Authority | Familiarity (Cs), judgment basis (Ca), authority coefficient (Cr) | Partial | 2.2 Level of Engagement and Authority of the Experts |
| 20c | Coordination | Coefficient of Variation (CV) and Kendall's W | Yes | 2.3 Degree of Coordination of Expert Opinions |
| 20d | Central Tendency | Mean importance score, full-score rate, CV | Partial | 2.4 Results of the Expert Consultation; Table 5; Supplementary Table S3 |
| 20e | Other Indicators | Weight coefficients, consistency coefficients, etc. | Yes | 2.4 Results of the Expert Consultation; Table 5; Supplementary Table S3 |
| 21 | Overall Process | Reasons for stopping, outlier handling, changes per round, flowchart | Yes | 2.4 Results of the Expert Consultation |
| 22a | Validity & Reliability | Validity and reliability analysis of consensus | Yes | 3.1 Methodological Rigor of the Indicator System |
| 22b | Theoretical Meaning | Theoretical implications and application scope | Yes | 3.2 Content Analysis: Comprehensiveness, Specialty Specificity, and Critical Comparison |
| 22c | Translation to Practice | Translation into practice and recommendations | Yes | 3.3 Feasibility and Practicality of the Indicator System  3.4 Implications for Nursing Management |
| 22d | Outlier Handling | Outlier handling and differential opinion explanation | Yes | 1.3.3 Implementation of the Expert Consultation |
| 23a | Limitation: Experts | Limitations of expert selection and number | Yes | 3.5 Limitations of the Study |
| 23b | Limitation: Data | Limitations of reported data | Yes | 3.5 Limitations of the Study |
| 23c | Other Limitations | Other potential limitations | Yes | 3.5 Limitations of the Study |
| 24 | Future Research | Future research directions | Yes | 3.6 Conclusion and Future Perspectives |
| 25a | Dissemination to Experts | Whether results were shared with expert panel | No | — |
| 25b | Publication | Dissemination via journal or health authorities | Yes | — |
| 26a | Reference Appropriateness | Appropriate reference citation | Yes | References |
| 26b | Recent Key References | Recent (3–5 years) key Chinese/English references | Yes | References |

Reference: Liu XH, Zhou Q, Cheng SZ, Wu YX, Liu Y. Development and interpretation of a reporting checklist for Delphi technique in clinical research papers [in Chinese]. Chin J Mod Nurs. (2024) 30(5):616–24. doi: 10.3760/cma.j.cn115682-20230914-01074
